# Larvicidal Activity of *Nerium oleander* against Larvae West Nile Vector Mosquito *Culex pipiens* (Diptera: Culicidae)

**DOI:** 10.1155/2015/943060

**Published:** 2015-11-11

**Authors:** Fouad El-Akhal, Raja Guemmouh, Yassine Ez Zoubi, Abdelhakim El Ouali Lalami

**Affiliations:** ^1^Regional Diagnostic Laboratory of Epidemiological and Environmental Hearth, Regional Health Directorate, EL Ghassani Hospital, 30000 Fez, Morocco; ^2^Sidi Mohamed Ben Abdellah University, Faculty of Sciences Dhar El Mahraz, Laboratory of Biotechnology and Preservation of Natural Resources, 30000 Fez, Morocco; ^3^Laboratory of Phytochemistry, National Institute of Medicinal and Aromatic Plants, 34000 Taounate, Morocco; ^4^Institute of Nursing Professions and Health Techniques, EL Ghassani Hospital, 30000 Fez, Morocco

## Abstract

*Background*. Outbreaks of the West Nile virus infection were reported in Morocco in 1996, 2003, and 2010.* Culex pipiens* was strongly suspected as the vector responsible for transmission. In the North center of Morocco, this species has developed resistance to synthetic insecticides. There is an urgent need to find alternatives to the insecticides as natural biocides. *Objective*. In this work, the insecticidal activity of the extract of the local plant *Nerium oleander*, which has never been tested before in the North center of Morocco, was studied on larval stages 3 and 4 of *Culex pipiens*.* Methods*. Biological tests were realized according to a methodology inspired from standard World Health Organization protocol. The mortality values were determined after 24 h of exposure and LC_50_ and LC_90_ values were calculated. *Results*. The extract had toxic effects on the larvae of culicid mosquitoes. The ethanolic extract of *Nerium oleander* applied against the larvae of *Culex pipiens* has given the lethal concentrations LC_50_ and LC_90_ in the order of 57.57 mg/mL and 166.35 mg/mL, respectively. *Conclusion*. This investigation indicates that *N. oleander* could serve as a potential larvicidal, effective natural biocide against mosquito larvae, particularly *Culex pipiens*.

## 1. Introduction

The diseases vectored by mosquitoes continue to be a major cause of illnesses and death worldwide [1, 2]. Malaria, filariasis, Japanese encephalitis, and dengue fever are the most apparent diseases (parasitic and viral) vectored by mosquitoes of the genera* Anopheles*,* Culex* and* Aedes* [3, 4].

In the years 1996, 2003, and 2010 and according to a research, outbreaks of West Nile virus infection were reported in Morocco [5, 6]. Moreover, the study stated that the* Culex pipiens* (*C. pipiens*) was strongly suspected as the vector responsible for transmission [5–8]. The intensity of the transmission depends on factors linked to the parasite, the vector, the host human, and the environment.

Due to the lack of awareness among people, early detection and complete treatment of these diseases were very difficult [9]. One of the available methods for controlling the mosquitoes is the use of synthetic insecticides, these latter adversely affecting the environment by contaminating the air, water, and soil [10].

In the North center of Morocco, the species* C. pipiens* has developed resistance to the synthetic insecticide: Temephos, which is usually used in an antilarval fight [11]. Let us note that this species* C. pipiens* has developed equal resistance on other insecticides as Malathion, Fenthion, and Fenitrothion (unpublished data). Therefore, there is an urgent need to find alternatives to the insecticides, as natural herbal biocides.

Different parts of plants contain a complex of chemicals with a unique biological activity. The hydroethanol extract of plants and their components are widely used in the prevention and treatment of some human diseases. Various extracts of plants have been also documented to exhibit acute toxic effects against insects, including mosquitoes.* Nerium oleander* (*N. oleander*) is flowering shrub of Dogbane family. It is popularly used as an ornamental plant [12]. Throughout history, this plant has been used in medicine, as an antibacterial, anti-inflammatory, and antinociceptive compound [13].

This study has been carried out with the aim to assess the larvicidal activity of hydroethanolic extract of* N. oleander* on* C. pipiens*. The insecticidal activity of* N. oleander* plant against* C. pipiens* has never been studied before in the North center of Morocco.

## 2. Materials and Methods

### 2.1. Crop Plants and Ultrasound-Assisted Extraction

A sample collection (leaves, stems and roots) of a local plant (*N. oleander*) was conducted in April (2014) at the mountain of Timezgana falling within the rural community of Timezgana (area of Taounate, North center of Morocco) to an approximate altitude of 800 m.

In a 500 mL beaker, 20 g of a dried plant powder was mixed with 150 mL of hexane. The beaker was set in a Sonicator brand “ELMA” a frequency of 35 kHz for 45 min, with a temperature of 25°C. The extract was filtered through Whatman paper and the recovered solvent was rejected. Drying the powder in a plant incubator at a temperature of 40°C for 30 mins, the powder was reextracted again with ethanol at 80% for 45 min under the same conditions. The final extract was recuperated from the mixture (ethanol/water) after filtration by Whatman paper and evaporation under vacuum at 40°C on a rotary evaporator [14].

### 2.2. Phytochemical Screening

The phytochemical constituents existing in the hydroethanolics are tannins, flavonoids, sterols, terpenes, triterpenes, coumarins, leucoanthocyanins, and mucilages, obtained using a simple qualitative analysis method, as described in the study [15, 16].

### 2.3. Characteristics of Larval Site

The collection of larvae of* C. pipiens* was performed in a breeding site located in the urban area of the city of Fez, called Grand Canal (402 m altitude, 30°03′37′′ N and 5°08′35′′ E). This site, originating from a hot spring, is characterized by a very high density of Culicidae larvae. The warm water from a thermal spring called Ain Lah promotes the proliferation of larvae of* C. pipiens*.

### 2.4. Collecting Larvae of C. pipiens

Larvae were collected using a rectangular plastic tray that was inclined 45° with respect to the water surface; the resulting tension force attracts the plate to the larvae. The larvae gathered were maintained in breeding in rectangular trays at an average temperature of 21.7°C ± 2°C in the Entomology Unit at the Regional Diagnostic Laboratory Epidemiological and Environmental Health (RDLEH) falling within Regional Health Directorate of Fez.

### 2.5. Identification of Larvae

The identification of morphological characters of larvae has been determined using the Moroccan key of identification of Culicidae [17] and the identification software of mosquitoes of the Mediterranean Africa [18].

### 2.6. Protocol of Larval Susceptibility Testing

The susceptibility tests were carried out in accordance with the standard protocol developed by WHO in 2005 [19]. From the initial extract (100 mg/mL stock solution) of plant, concentrations of 20, 40, 60, 80, 100, 120, 140, and 160 mg/mL were prepared. Preliminary experiments were used to select a range of concentrations for the tests previously mentioned. 1 mL of each solution prepared was placed in beakers containing 99 mL of distilled water in contact with 20 larvae of stages 3 and 4; the same number of larvae was placed in a beaker containing 99 mL of distilled water plus 1 mL of ethanol. Three replicates were carried out for each dilution and for the control. After 24 hours of contact, we counted the living and dead larvae. The results of susceptibility testing were expressed in the percentage of mortality versus the concentration of plant extract used. If the percentage of mortality in control is greater than 5%, the percentage of mortality in larvae exposed to the extract shall be corrected by using Abbott's formula [20]:

(1) %  Mortality  Corrected=%  Mortality  Observed−%  Mortality  Control100−%  Mortality  Control×100.

If the control mortality exceeds 20%, the test is invalid and must be repeated.

### 2.7. Data Processing

For the data processing we used the log-probit analysis (Windl version 2.0) software developed by CIRAD-CA/MABIS [21]. The analysis of the averages and standard deviation was also performed by using the test of analysis of variance ANOVA.

## 3. Results

As it is illustrated in Table 1, the phytochemical screening of extract of* N. oleander* grown in North center of Morocco revealed a presence of flavonoids, sterols, terpenes, triterpenes, and coumarins components. However, tannins, leucoanthocyanins, and mucilages were not detected.

### 3.1. Variation in Mortality Rate

**Figure 1:**
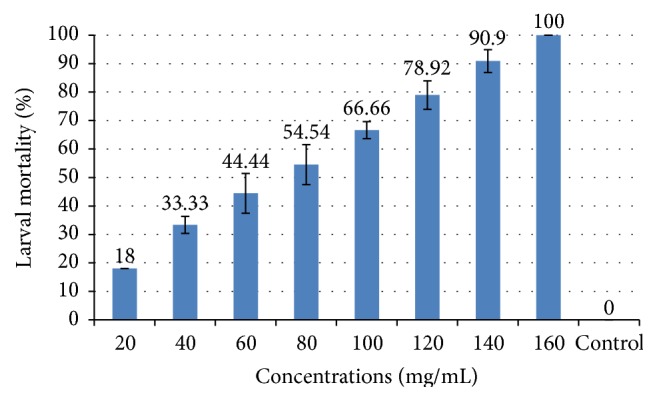
Percentage of mortality recorded in the test sensitivity by aqueous extract of a plant on* C. pipiens*.

The hydroethanolic extract of* N. oleander* is used. The mortality rate ranged between 18% and 100% (Figure 1). The lowest concentration necessary to achieve 100% mortality of larvae of* C. pipiens* was evaluated at 160 mg/mL.

### 3.2. LC_50_ and LC_90_ Lethal Concentrations

Figure 1 confirms the analysis performed to the order of effectiveness of aqueous extracts tested. The aqueous extract of* N. oleander* exhibits the lowest LC_50_ of 57.57 mg/mL (equation of the regression line: *Y* = −4.89649 + 2.78167*∗X*; calculated Chi^2^: 23.364) and LC_90_ = 166.35 mg/mL (Table 2).

## 4. Discussion

It is a noteworthy fact that plants have been evolving for over 400 million years and have developed protection mechanisms, such as repellents and even insecticidal effects, to defend themselves against insect attack. Many research papers have reported the efficiency of plant extracts against mosquito larvae [22–24].

The results of phytochemical screening of the extract of* N. oleander*, which we found are in accordance with other studies related to the Nerium family. The species of this family produce flavonoids, coumarins, and triterpenes [25, 26].

The larvicidal activity observed among extracts of* N. oleander* could be explained by the action or effect of phytochemical components: flavonoids, sterols, terpenes, triterpenes, and coumarins.

Flavonoids have a key role in stress response mechanisms in plants. The adaptive role of flavonoids in plant self-protection against bacterial, fungal, and viral diseases as well as insects starts to gain importance in the understanding of plant defense. Flavonoids, which act as antioxidants or enzyme inhibitors, are involved in photosynthesis and cellular energy transfer processes and may serve as precursors of toxic substances [27, 28] or have a pharmacological activity [27].

In Vellore City, India, the larvicidal activity of* N. oleander* was evaluated against* Culex* larvae; 43% of mortality was found from the concentration of 3% (30 mg/mL) during the 24-hour exposure [12]. In our work, this percentage of mortality was observed for a concentration of 60 mg/mL (which is the double of concentration found by the previous study).

In another work, Madhuri et al., in 2013 [29], found that* N. oleander* did not show any larvae mortality of* Culex* in 1% aqueous extract, whereas 100 ppm (0.1 mg/mL) of* N. oleander* had 74% mortality in 72 hours.

The results obtained by El-Sayed and Ghada in 2014 [30] indicated that diethyl ether extract of* N. oleander* leaves should reduce the population dynamics of* C. pipiens*, with LC_50_ of 10500 mg/L (10.5 mg/mL). In this study, we found 57.57 mg/mL for the LC_50_, which is five times more than the concentration obtained by El-Sayed and Ghada.

In the North West of Morocco, one preliminary evaluation study of the larvicidal activity of the* N. oleander* plant on fourth-stage larvae (L4) of the species* C. pipiens* was conducted. This study made by Aouinty et al. in 2006 [31] who exhibited that the aqueous extract of this plant has been ineffective in terms of toxicity with a calculated LC_50_ of about 3130 ± 310 mg/L (3.13 mg/mL).

Various factors as the environmental conditions, the extraction technique, the drying, the period and gathering sites, the agricultural practices, the plant age [32–35], the concentration of the extract, the concentration of its active components, or even factors regarding the mosquito can influence the performance, the physicochemical characteristics, and the chemical composition of the extract.

Thus, Fakoorziba et al. in 2015 [36], in southern Iran, concluded that there was a high to low lethal effect of extracts of* N. oleander* leaves against mosquito larvae (Diptera: Culicidae):* Anopheles stephensi* depending on the solvent used: chloroform, petroleum, benzene, water, and acetone.

Comparing our results with the work mentioned previously, we can deduce that the LC_50_ obtained by the larvicidal action of the* N. oleander* plant grown in North East of Morocco on the larvae of the* C. pipiens* is relatively effective. However, if we compare our LC_50_ (57.57 mg/mL = 57570 ppm) to that of a chemical larvicide, for example, Temephos which was found between 0.0065 and 0.0094 ppm against* C. pipiens* [11], we can deduce that our LC_50_ is far too high to be effective against mosquito larvae. Consequently, the plant extract should not really be considered an effective larvicide against mosquito.

That is why, the extracts of the aqueous phase of the* N. oleander* grown in North center of Morocco should be subjected to separation to isolate and concentrate the active substances, which certainly can present much lower LC_50_ and would be valued as an alternative insecticide.

On the other hand, several studies have reported that all parts of the oleander plant, including the sap, dried or boiled, are poisonous to humans, animals, fish, birds, and in particular certain insects [37–40]. Indeed, the leaves contain a mixture of a very toxic cardiac glycosides of cardenolides like oleandrin, oleandrigenin, digoxin, digitonin, digitoxigenin, nerizoside, neritaloside, and odoroside [41, 42].

## 5. Conclusion

This study concluded that the hydroethanolic extract of* N. oleander* possesses larvicidal activity with values of LC_50_ and LC_90_, of 57.57 mg/mL and 166.35 mg/mL, respectively. The larvicidal activity of hydroethanolic extract of* N. oleander* on* C. pipiens* could be due to the major components. This would include the flavonoids, the sterols, the terpenes, the triterpenes, and the coumarins.

This plant can be used as a biological remedy to control the mosquito population in the locality at risk, particularly the* Culex* mosquito which possesses great menace of deadly disease transmission. Further studies on isolation of active constituents from the extract and their biological activity on culicid mosquito are under investigation.

## Figures and Tables

**Table 1:** Phytochemical screening of hydroethanolic extract of plant *N. oleander*.

Plant	Tannins	Flavonoids	Sterols and Terpenes	Triterpenes	Coumarins	Leucaonthocyans	Mucilages
*Nerium oleander*	−	**+**	**+**	**+**	**+**	−	−

(+) = present; (−) = absent chemical.

**Table 2:** Concentrations LC_50_ and LC_90_ lethal larvae of *C. pipiens* after 24 hours of exposure.

Plant	LC_50_ (mg/mL) (Ll-Ul)^*∗*^	LC_90_ (mg/mL) (Ll-Ul)^*∗*^
*N. oleander*	57.57 (36.75–75.92)	166.35 (118.19–365.79)

^*∗*^Ll-Ul: lower limit-upper limit.
